# 522 Safe Use of Thrombolytic Therapy for Severe Frostbite in Pediatric Patients

**DOI:** 10.1093/jbcr/irad045.119

**Published:** 2023-05-15

**Authors:** Melanie McCormick, Sam Miotke, Alexandra Lacey

**Affiliations:** University of Minnesota, Minneapolis, Minnesota; Regions Hospital, Saint Paul, Minnesota; HealthPartners, Saint Paul, Minnesota; University of Minnesota, Minneapolis, Minnesota; Regions Hospital, Saint Paul, Minnesota; HealthPartners, Saint Paul, Minnesota; University of Minnesota, Minneapolis, Minnesota; Regions Hospital, Saint Paul, Minnesota; HealthPartners, Saint Paul, Minnesota

## Abstract

**Introduction:**

Frostbite in children is very rare with few citations in the literature. Thrombolytic therapy is commonly used to salvage threatened tissue in frostbitten adults, but there is little to no data on the use of such therapy in children. The aim of this case series is to advance our understanding of pediatric frostbite by describing risk factors and outcomes, as well as commenting on the safety of thrombolytic therapy in this population. To our knowledge, thrombolytic therapy has not been studied in children, and this is the largest case series of pediatric frostbite to date.

**Methods:**

This is a retrospective, single-center study reviewing cases of pediatric (< 14 years) frostbite from 2006-2022. Thirteen patients (6 males and 7 females) were found to fit inclusion criteria, and data was collected from the electronic medical record.

**Results:**

A total of thirteen cases were reviewed. Nine patients were managed conservatively with diligent local wound cares, while four were prescribed thrombolytic therapy in an attempt at tissue salvage. Two patients underwent intravenous thrombolytic therapy, and two underwent intra-arterial thrombolytic therapy; there were no complications in either group. We also reviewed patient demographics. Thirty-eight percent of patients were documented as Black or African American, 23% Asian, 23% Caucasian, and 8% Hispanic or Latino. The remainder were unlisted. Notably, 31% of the children had trouble with communication (whether foreign speaking or non-verbal). None of the patients had a positive EtOH or urine drug screen. All of the children were poorly supervised when the frostbite occurred. Outcomes were as follows: 69% were completely healed upon follow up, 23% had very severe frostbite injury upon arrival with significant tissue loss likely requiring amputation (but did not return for follow up), and 8% never followed up.

**Conclusions:**

The use of thrombolytics for the treatment of frostbite injury appears to be safe in children. We have found that risk factors for frostbite in this population include an inability to communicate and poor supervision at the time of injury.

**Applicability of Research to Practice:**

We have been able to safely treat children presenting with severe frostbite injury with thrombolytic therapy. This work is highly applicable to practice. Specifically, the ability to administer intravenous thrombolytic therapy is nearly ubiquitous. This can be done at referring hospitals if there are issues with inclement weather or transportation that would delay transfer to a verified burn center.

